# Translating Scientific Advances in the AOP Framework to Decision Making for Nanomaterials

**DOI:** 10.3390/nano10061229

**Published:** 2020-06-24

**Authors:** James D. Ede, Vladimir Lobaskin, Ulla Vogel, Iseult Lynch, Sabina Halappanavar, Shareen H. Doak, Megan G. Roberts, Jo Anne Shatkin

**Affiliations:** 1 Vireo Advisors, LLC, Boston, MA 02130, USA; jede@vireoadvisors.com; 2 School of Physics, University College Dublin, Belfield, Dublin 4, Ireland; vladimir.lobaskin@ucd.ie; 3 National Research Centre for the Working Environment, DK-2100 Copenhagen, Denmark; UBV@nfa.dk; 4 School of Geography, Earth and Environmental Sciences, University of Birmingham, Edgbaston, Birmingham B15 2TT, UK; I.Lynch@bham.ac.uk; 5 Environmental Health Science and Research Bureau, Health Canada, Ottawa, ON K1A 0K9, Canada; sabina.halappanavar@hc-sc.gc.ca; 6 Institute of Life Sciences, Swansea University Medical School, Singleton Park, Swansea SA2 8PP, UK; s.h.doak@swansea.ac.uk; 7 Department of Chemistry, University of Toronto, Toronto, ON M5S 3H6, Canada; mroberts@vireoadvisors.com

**Keywords:** adverse outcome pathway, nanomaterials, decision making, risk assessment

## Abstract

Much of the current innovation in advanced materials is occurring at the nanoscale, specifically in manufactured nanomaterials (MNs). MNs display unique attributes and behaviors, and may be biologically and physically unique, making them valuable across a wide range of applications. However, as the number, diversity and complexity of MNs coming to market continue to grow, assessing their health and environmental risks with traditional animal testing approaches is too time- and cost-intensive to be practical, and is undesirable for ethical reasons. New approaches are needed that meet current requirements for regulatory risk assessment while reducing reliance on animal testing and enabling safer-by-design product development strategies to be implemented. The adverse outcome pathway (AOP) framework presents a sound model for the advancement of MN decision making. Yet, there are currently gaps in technical and policy aspects of AOPs that hinder the adoption and use for MN risk assessment and regulatory decision making. This review outlines the current status and next steps for the development and use of the AOP framework in decision making regarding the safety of MNs. Opportunities and challenges are identified concerning the advancement and adoption of AOPs as part of an integrated approach to testing and assessing (IATA) MNs, as are specific actions proposed to advance the development, use and acceptance of the AOP framework and associated testing strategies for MN risk assessment and decision making. The intention of this review is to reflect the views of a diversity of stakeholders including experts, researchers, policymakers, regulators, risk assessors and industry representatives on the current status, needs and requirements to facilitate the future use of AOPs in MN risk assessment. It incorporates the views and feedback of experts that participated in two workshops hosted as part of an Organization for Economic Cooperation and Development (OECD) Working Party on Manufactured Nanomaterials (WPMN) project titled, “Advancing AOP Development for Nanomaterial Risk Assessment and Categorization”, as well as input from several EU-funded nanosafety research consortia.

## 1. Introduction

Manufactured nanomaterials (MNs), as well as other advanced and emerging materials (e.g., composites incorporating MNs), offer significant benefits to consumers, including technological innovation, improved product performance and more sustainable alternatives to current technologies [1]. However, safety evaluations of these materials are lagging due to testing challenges, the number, diversity and complexity of MNs coming to market, and the fact that the requirements of regulatory organizations are evolving as understanding of the technology develops. Currently, toxicological assessments of MNs require individual assessments of health and environmental endpoints using traditional animal testing methods, an approach that is too time- and cost-intensive to be practical, as well as being ethically concerning. Today’s toxicity testing landscape is facing pressure to enhance transparency and incorporate up-to-date scientific understanding in risk assessments and related decision-making. Regulators must accurately assess the safety of tens of thousands of new and existing chemical substances, but the resources allotted for safety assessments are often static or even decreasing [2]. Policy is shifting globally toward the reduction and eventual elimination of animal studies, favoring alternative methods for all chemicals (e.g., in the EU [3] and the US [4].

The science required to address the technical challenges of transitioning to alternative (nonanimal) toxicity testing is progressing, but efforts are needed to incorporate these developments into decision making and to guide the science to address data and methodological gaps hindering regulatory risk assessment [5]. Understanding the mechanisms-of-action underlying adverse health effects is an essential prerequisite for the development of alternative tests for decision-making [6]. Over the past several decades, significant advances have been made in toxicological evaluations that utilize in silico, in chemico, in vitro and ex vivo approaches to predict the hazards of new chemicals, including MNs, without animal testing [2]. Improvements in computational capabilities have made in silico modeling experiments more accurate and the ability to model complex system responses more feasible. Advances in genomics and proteomics have improved our understanding of how changes in gene and protein expression may indicate the biological events that lead to adverse outcomes in organisms, and bioinformatics has led to the development of methods and tools to analyze the enormous amounts of data generated through these approaches. In vitro models have advanced, improving the characterization of complex biological systems. These new toxicological tools evaluate biological responses on the molecular, subcellular, cellular and tissue levels, and offer risk assessors more mechanistic information than ever before.

Currently, however, few in vitro and ex vivo tests are accepted for use in regulatory decision-making, largely due to a lack of formal validation. The widely acknowledged challenges for alternative testing approaches apply to conventional chemicals and MNs alike, but these issues are further complicated for MNs. MN toxicity testing is challenging, in part because of their unique physical and chemical properties. MN dose-response relationships may differ from those of conventional materials. Dose-response relationships for conventional materials are based on dose in mass terms, and do not account for the potentially enhanced toxicity caused by the particle aspects and increased specific surface area of MNs. In addition, identifying groups for categorization based on the biological effects arising from small changes in the MN physical and chemical characteristics is not easy. MN toxicity has been shown to be influenced by several physical–chemical properties, including dissolution rate (ion release), electronic band gap, aspect ratio, dispersibility in solution, contaminants, particle size and surface chemistry. These physical–chemical characteristics are all interconnected, and determining the contribution of a single parameter to toxicity remains difficult. Regulatory agencies have recognized that more efficient testing strategies are needed for MNs, but that they also require further development and verification before incorporation into regulatory testing guidance documents. How to incorporate and use in vitro and ex vivo data to assess the risks of MNs and other new chemicals often remains unclear; the translation of these approaches from experimental to regulatory applications remains difficult.

The dual challenge of advancing MN risk assessment while reducing costs and reliance on animal testing presents an opportunity to develop smarter approaches to screening which prioritize novel nanoscale materials. Doing so will require a coordinated response that adopts science into decision making processes and bridges current knowledge gaps. The AOP framework is an evidence-based approach that is expected to aid in the resolution of several twenty-first century challenges regarding chemical and nano-specific safety assessments [7,8,9]. Significant progress has been made in AOP development, application and use over the last decade, including methods, resources and tools to adopt AOPs as part of an integrated approach to testing and assessment (IATA) that utilizes alternative testing data for decision making. The AOP framework offers a systematic, mechanistic approach to assess MN-induced risks with data from nonanimal testing, thereby both enabling the development of cost- and time-efficient testing strategies and the implementation of safe-by-design approaches, and reducing reliance on animal testing. Although the science behind AOPs has advanced considerably, a number of barriers remain regarding the adoption of AOPs for risk assessments of MNs. An international, coordinated effort is needed to address the technical and translational issues in order to realize the potential of AOPs for evidence-based decision making.

Recognizing this potential, a number of initiatives are developing MN-relevant AOPs. Notably, the EU and the OECD are both spearheading major initiatives, including the EU H2020 SmartNanoTox program and related projects, as well as the OECD AOP Development Programme. The promise of the AOP framework is confirmed in MN-specific guidance documents, such as the European Food Safety Authority (EFSA), “Guidance on risk assessment of the application of nanoscience and nanotechnologies in the food and feed chain [10],” which states that “The Organization for Economic Co-operation and Development now also explores Integrated Approach to Testing and Assessment (IATA) and promotes the use of AOPs to build risk assessment, while assessing all the existing data.”

At the same time, however, more work is needed. The EFSA guidance, for instance, goes on to say that “The developments in efficient testing strategies and AOPs for nanomaterials are highly acknowledged, though they need further development and verification before incorporation into guidance documents can be considered.”

This review describes the AOP framework and its current status in greater detail, outlines challenges facing its advancement and adoption, and recommends specific actions needed to mature the concept and advance its use for decision making which is specific to MNs. The recommendations are organized into short-, medium- and long-term actions.

## 2. Materials and Methods

This review reflects the views of a diversity of stakeholders including experts, researchers, policymakers, regulators, risk assessors and industry representatives on the current status, needs and requirements to facilitate future use of AOPs in MN risk assessment. It incorporates the views and feedback of experts that participated in two workshops hosted as part of an Organization for Economic Cooperation and Development (OECD) Working Party on Manufactured Nanomaterials (WPMN) project titled, “Advancing AOP Development for Nanomaterial Risk Assessment and Categorization (NanoAOP project)”. The first workshop was hosted on 19 September, 2018 during the NanoToxicology conference in Neuss, Germany. The second workshop was held on 11 September, 2019 at the OECD Conference Centre in Boulogne-Billancourt, France, and presented the results of three related projects addressing the development and use of AOPs for MN risk assessment: (1) the OECD WPMN NanoAOP project; (2) the Horizon 2020 (H2020) project SmartNanoTox; and (3) the H2020 project PATROLS.

Over 40 workshop participants from Canada, Chile, Denmark, Finland, France, Germany, Ireland, Italy, Japan, Korea, Netherlands, Switzerland, Turkey, U.K. and the U.S. were divided into three groups, with discussions in each centered around one of three central themes (Table 1).

Among other experts, participants weighed in on an earlier draft of this work and related deliverables [11], in terms of the needs and priorities regarding the use of AOPs in risk assessment. Rapporteurs from breakout groups recorded the responses, comments and recommendations from the experts, which were collated into a workshop summary report to be published in 2020/2021.

## 3. Overview of the AOP Framework and Current Status

### 3.1. Overview

AOPs clarify relationships across biological levels of organization (including molecular, subcellular, cellular, tissue, organ, organism and whole populations), using cause-and-effect relationships to connect molecular initiating events (MIE) to adverse outcomes (AO). AOs are negative biological consequences resulting from chemical exposure; they are typically measured at higher levels of biological organization and are important for regulatory decision making, e.g., concerning issues of human health (organ or organism) or environmental endpoints (organism or populations). The pathways connecting MIEs and AOs are defined by key events (KEs) which represent measurable biological changes, and key event relationships (KERs), i.e., the directed, predictive relationships among those KEs. AOPs are the units of development for the AOP framework, and represent a single, nonbranching sequence of KEs, linked by KERs, connecting a single MIE to a single AO [12].

**Figure 1:**
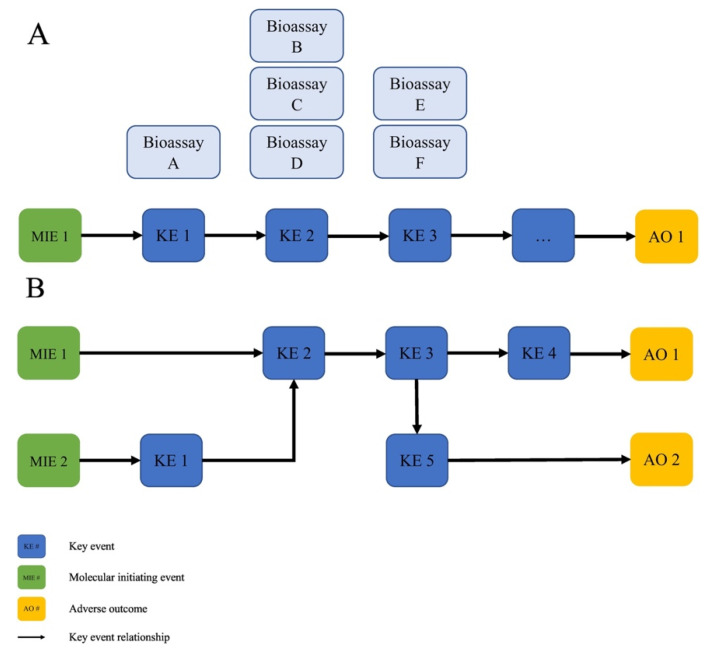
(**A**) Generalized AOP showing the relationship between MIE, KEs and AOs, and the KERs that connect them. Bioassays targeting the MIE and KEs in an AOP are characterized or developed as part of an IATA. (**B**) AOPs can form interlinked networks based on overlapping MIEs, KEs and AOs that better capture the complex biology of disease processes. (From [11]).

Figure 1 gives an example of a generalized AOP and its components (Panel A), and shows how AOPs can form interlinked networks based on overlapping MIEs, KEs and AOs (Panel B) that represent the complex biology underlying disease processes [11]. Bioassays targeting the MIE and KEs in an AOP were developed, characterized and used as endpoints as part of an IATA. The AOP framework defines this entire conceptual approach that assembles and organizes mechanistic knowledge to communicate causal links between biological perturbations and adverse health outcomes which are meaningful to chemical risk assessments and regulatory decision making [2,13].

To help accelerate AOP development, strategies, principles and best practices have been established to help scientists, regulators and decision makers understand and contribute to the AOP framework. In 2014, Villeneuve et al. proposed a set of five core principles to guide AOP development [14]; these are highlighted in Box 1.

Box 1 **Core Tenets of AOPs [14].**

1. AOPs are not chemical-specific. Specificity limits the predictive utility of AOPs for new substances.
2. AOPs are designed with modular units. These components should be reusable to enhance flexibility, and they should be designed to accommodate differing levels of detail based on evidence.
3. AOPs are a unit of development. An individual AOP is defined as a single, nonbranching sequence of KEs, linked by KERs, connecting a single MIE to a single AO. This structure reduces the complexity, and is a practical unit for development and evaluation.
4. AOPs form networks. Multiple AOPs, sharing one or more common KE or KER, form networks that more realistically represent the complexity of biological systems needed to make accurate biological predictions of adverse toxicological outcomes.
5. AOPs should be continuously updated. New research should be used to inform and refine existing AOPs.

### 3.2. Current Status

**Figure 2:**
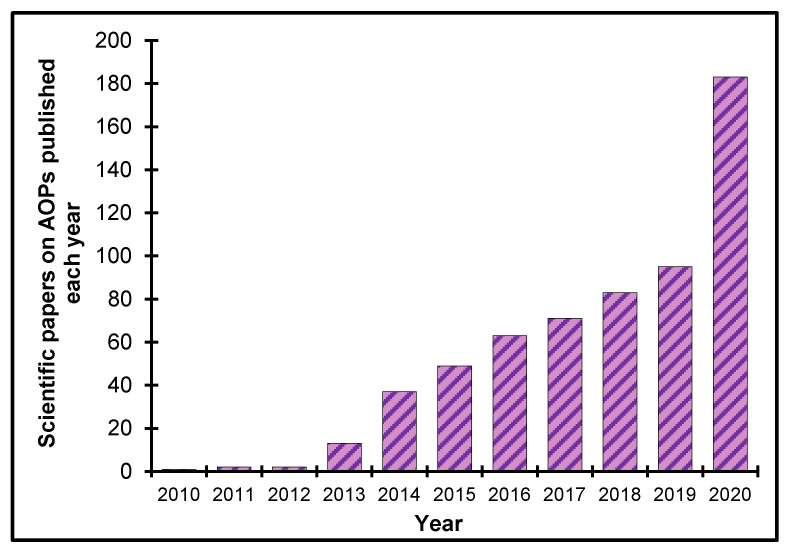
Scientific papers on AOPs published annually, 2010–2019. PubMed was searched for published papers containing the text words “adverse outcome pathway” on 22 May, 2020. The result for 2020 is estimated given the number of publications that have been published per month.

Tremendous progress in AOP research and development has occurred since AOPs were first described in 2010 [13]. A survey of papers published annually on the topic from 2010–2019 indicated exponential growth, with almost 200 publications expected in 2020 based on a PubMed literature search. This growth suggests that the AOP concept is gaining widespread traction and acceptance in the academic community (Figure 2 [15]).

A number of efforts worldwide are contributing to AOP development; among these, the OECD is spearheading one of the largest. The OECD AOP Development Programme was started in 2012, and is overseen by the Extended Advisory Group on Molecular Screening and Toxicogenomics (EAGMST). Its goal is to develop, review and officially endorse AOPs. AOP development is based on voluntary contributions from member countries and stakeholders. It involves an internal review within EAGMST to ensure compliance with AOP principles, as well as an external review by subject matter experts to assess the scientific merit of the proposed pathway. There are currently nine OECD-endorsed AOPs, seven that have received approved status, fourteen under review (indicating that they are at a late stage of the endorsement process), and twenty-four proposals under active development.

**Figure 3:**
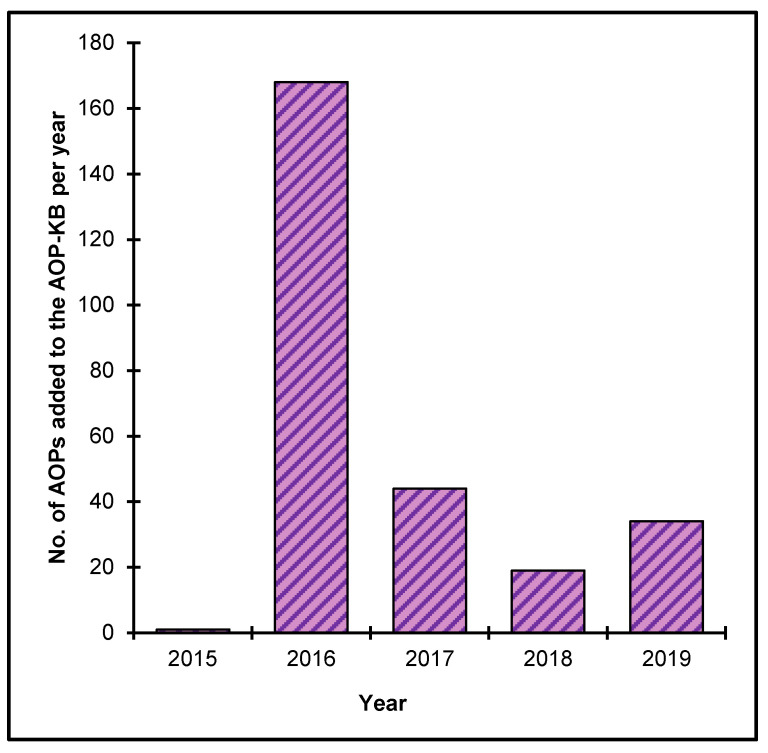
AOPs created in the AOP-KB from 2015–2019 (until May 22, 2020).

The OECD, together with contributions from the U.S. EPA, the European Commission’s Joint Research Centre and the U.S. Army Engineer Research and Development Center has also led the development of the AOP Knowledge Base (AOP-KB). Launched in 2014, this web-based tool consists of five modules to enable and promote the development and application of AOPs: the e.AOP.Portal, the AOP-Wiki, the Effectopedia, the AOP Xplorer and the Intermediate Effects Database [12]. The AOP-Wiki serves as the primary repository for qualitative AOPs developed (including those endorsed by the OECD) or under development. It is intended to foster collaboration among various stakeholders contributing to AOP development following the standard OECD principles for developing and assessing AOPs [16]. The AOP Wiki currently contains more than 200 AOPs, including more than 2000 defined KEs (Figure 3).

In addition to these efforts, various stakeholders have supported projects aimed at evaluating and promoting the development of AOPs for MNs. The EU, through its H2020 initiative, has supported several projects focused on the development and application of AOPs for MNs; these include SmartNanoTox (Smart Tools for Gauging Nano Hazards) and PATROLS (Physiologically Anchored Tools for Realistic nanOmateriaL hazard aSsessment) [9,14]. Since 2016, the OECD Working Party on Manufactured Nanomaterials (WPMN) has included in its program the project *Advancing Adverse Outcome Pathway (AOP) Development for Nanomaterial Risk Assessment and Categorisation* [NanoAOP Project; ENV/CHEM/NANO(2017)5]. These projects and their outcomes and contributions to AOP development are discussed in Section 6.

## 4. Potential Applications of AOPs

Progress in AOP development has given rise to a range of potential applications. In chemical risk assessment, the AOP framework is intended to guide and develop IATA. IATA is an approach to characterizing the hazard of chemicals that integrates analyses of existing information with the generation of new information through targeted testing strategies [12]. From a risk assessment perspective, the AOP framework provides:

- A structured framework to evaluate existing information available for a chemical of interest; potential sources include in chemico, in silico, in vitro, ex vivo, in vivo and ‘omics’ data;
- A way to identify data gaps and efficiently generate missing information to increase confidence in decision making and assessments of risk;
- A framework to apply an iterative approach until sufficient information is gathered for decision making.

Within this context, the AOP framework has several specific applications and benefits to improve risk assessment and decision making for chemicals; these are summarized in Table 2 [12,15,17]. Of particular interest, the AOP concept offers a systematic, mechanistic framework to develop, assess, use and interpret alternative testing strategies for chemical risk assessment and decision-making, thereby reducing reliance on new substance testing. Applications in chemical safety assessment are especially useful to regulatory toxicologists, risk assessors and risk managers, as well as to industry stakeholders responsible for product stewardship and compliance with regulatory requirements [2].

One area of proposed application of AOPs is MN risk assessment and decision-making. Because of their particulate aspects, MNs do not necessarily display classical dose-response relationships, and their toxicity is not always predictable from chemical substance models. Moreover, the biological effects of small changes in their physical and chemical makeup are not easily predicted with today’s risk assessment toolbox and models. The benefits of a framework that is better suited to assessing the impacts of such MN modifications can apply to regulators, researchers and product developers. One of the essential components of the mechanistic representation of MN-induced outcomes provided by AOPs is the MIE, that triggers the pathway, and KEs, that can be directly steered by MN interactions with biomolecules. The molecular level description of the initiating event and the underlying physical interaction make it possible to relate the physicochemical properties of the MN to the probability of a MIE via quantitative structure– or property–activity relationships (QSAR/QSPR). These relationships help to identify the MN properties of concern, and thus, enable the consequent grouping of the MNs based on their ability to induce a MIE/AOP. The main benefit of these relationships is the replacement of biological testing with in silico or in vitro screening.

While not the focus of this discussion, the AOP framework has other potential applications that extend beyond risk assessment [2]. AOPs provide utility in product discovery and development, especially in the pharmaceutical and agrochemical industries; here, they can support preclinical safety assessments to identify compounds, early in their development, which are potentially harmful to human, animal or environmental health. In medicine and health, clinicians and researchers can use AOP knowledge to understand disease pathways across multiple biological levels, informing prevention, diagnosis and treatment efforts. Other stakeholders that may benefit from AOP applications include academics (who may benefit from a unifying framework to increase the real-world impact of their work) and nongovernmental organizations (NGOs), including animal welfare and environmental NGOs. As mentioned, several focus areas in the proposed Horizon Europe Strategic Plan [18] could benefit from advancements and the adoption of AOPs in decision making.

## 5. Recent Progress with AOPs for Nanosafety

Projects worldwide are focusing on meeting the challenges of AOP development and promoting the use of AOPs for decision making. Below, projects contributing to the development, use and adoption of AOPs for MNs are highlighted and summarized.

### 5.1. Progress in AOP Development for Manufactured Nanomaterials

Inhalation exposure to powdered MNs, especially during manufacture and handling, is a highly relevant risk scenario [20]. Some studies have documented significant AOs such as fibrosis and cancer following exposure to certain forms of MNs (e.g., carbon nanotubes [21]); however, considerable uncertainty about the physical and chemical properties influencing these outcomes remains. In vivo inhalation experiments to characterize the hazard of MNs are challenging and resource-intensive. Of all standardized toxicity testing, inhalation studies are some of the most expensive to commission and complete. To simulate realistic exposure, animals have to be exposed to low dose aerosols over long periods of time (up to 2 years). Generating MN aerosols can be difficult (e.g., many are viscosity modifiers), and often, alternative exposure methods such as pharyngeal aspiration or intratracheal instillation must be used that deliver high bolus doses over short time frames [20]. The AOP framework, as part of an IATA, offers a more time- and cost-efficient alternative approach to assessing the potential risks from inhaled MNs; however, development and verification of the pathway is necessary before such an approach can be used for decision making.

The SmartNanoTox project is using results from in vivo, in vitro and in silico research to develop AOPs for adverse pulmonary effects following MN exposure. The effort is using representative sets of MNs to identify critical KEs and KERs to construct AOPs and relate them to interactions at the bio-nano interface [9]. Using the data generated from various KEs and KERs along the AOP, SmartNanoTox aims to develop quantitative structure–activity relationships (QSARs) to enable predictions of whether a MN can trigger an AOP and lead to an AO, and to enable the grouping, categorization and read-across of MNs for these endpoints [22,23]. The PATROLS project has collated and generated data to establish AOPs for both lung and liver inflammation, fibrosis and cancer AOs following MN exposure. The purpose of this activity was, based on our understanding of AOPs, to identify biomarkers which are suitable for long-term MN exposure effects that could be applied in short-term in vitro assays to develop more targeted nonanimal hazard testing strategies with potentially higher predictive value. NanoCommons has implemented a tool for predicting occurrences of MIEs using differentially expressed genes/proteins from high-throughput experiments to calculate a prioritized list of MIEs with identified biological processes, while NanoSolveIT is developing the concept of a nanomaterial fingerprint that captures the key physicochemical, biomolecular and interactional features of a MN that are predictive of its toxicity.

Other related efforts are developing data, methods and tools which are useful for AOP development [24,25,26]. For example, the EU’s NanoSolveIT project is developing: (i) innovative modelling techniques and tools for nanoinformatics; (ii) an IATA to identify the specific characteristics of MNs that are responsible for adverse effects on human health or the environment; and (iii) in silico methods, models and tools which are useful for AOP development. The grouping strategies, data and methods in the H2020 projects GRACIOUS and NanoReg 2 are being incorporated into IATA [27]. Similarly, NanoCommons aims to deliver a nanoinformatics research infrastructure including a database to facilitate the reuse of existing nanosafety data, and in silico tools for analyses and predictions of MN impacts, including tools to predict MIEs and AOPs. The Data and Knowledge on Nanomaterial (DaNa) project has compiled data on the applications of MNs and the current state of knowledge. The EU Cluster of Systems of Metadata for Official Statistics (COSMOS) is identifying common sets of metadata objects with standard definitions and methods to build better metadata repositories. NanoCommons, in collaboration with the National Cancer Institute working group on nanoinformatics, is supporting the development of metadata standards for nanosafety research, as described in another paper in this special issue [28]. All of these efforts can make significant contributions to AOP development.

### 5.2. Progress in the Application and Use of AOPs

One of the first AOPs officially endorsed by the OECD was the skin sensitization AOP (AOP No. 40 in the AOP-Wiki) [15]. Since its endorsement, mechanistic knowledge gained on skin sensitization has been used to develop and validate three standardized in vitro tests targeting the KEs in the AOP. These test guidelines have been published and are now accepted for regulatory use as a viable alternative to traditional animal testing for skin sensitization. The skin sensitization AOP is a success story, demonstrating how AOP development can help identify, promote development of, and validate alternative testing strategies for chemical risk assessment without the use of animals.

While significant progress has been made in AOP development, application and use over the last decade, for endpoints beyond skin sensitization, there is still a significant distance between the current reliance on, reduction of, and replacement of animal testing with nonanimal approaches for risk assessment and regulatory decision making. The OECD WPMN NanoAOP project is contributing to both the development and application of AOPs for MN risk assessment. The goal of the project was to develop a methodology to use existing nanotoxicology literature to support MN-relevant AOP development. While not developing an AOP, a case study outlines how the literature can be mined to identify and develop specific KEs to support AOP development.

As part of its outcomes, the OECD WPMN NanoAOP project convened two workshops to gain expert feedback on the current status, use and future needs for AOPs which are relevant to MNs in support of risk assessment. The consensus from experts is that currently, the primary applications of AOPs are (i) to support hazard identification; (ii) grouping, categorization and read-across; (iii) ranking and prioritizing MNs; (iv) the identification of novel biomarkers for alternative test method development; (v) for product development as part of a safer manufacturing approach; and (vi) together with ‘omics’ strategies, AOPs can be used to propose testing that could be predictive of AOs. The ultimate goal is to use AOPs as the basis for regulatory decision making for MNs; however, several challenges have been identified by experts that need to be overcome to advance the future use of AOPs for MN risk assessment (discussed in Section 7). Experts suggest that to ensure the relevance of AOP frameworks for regulatory decision making, development should proceed by first choosing an AO relevant for regulators, and then developing the pathway working backwards toward an MIE.

Several projects are contributing directly to the application and use of AOPs for MN risk assessment. As described, SmartNanoTox and PATROLS are developing in vitro models, methods and computational tools for MN hazard assessment targeting the KEs in developed AOPs. This is an important step to ensure that identified KEs in an AOP can be assessed with validated methods and tests, and thus, to support their inclusion in an IATA for MN risk assessment. SmartNanoTox is constructing simplified in vitro or in silico tests for the AOPs developed in their project, targeting identified MIEs and KEs for adverse respiratory outcomes from MN inhalation. PATROLS is focused on developing mechanism-based, nonanimal methods, models and computational tools for MN hazard characterization, targeting the KEs in established AOPs. This includes in silico hazard testing systems, in vitro human tissue models, ecotoxicology models and methods for MN characterization in biological systems.

## 6. Challenges in the Development and Application of AOPs

Substantial progress has been made toward the development and application of AOPs, but a number of challenges remain before their full potential can be realized. A global horizon scanning exercise to identify current challenges toward the regulatory adoption of the AOP framework is one of the largest efforts to advance AOP development and application [29]. The key findings from that effort are summarized below, as are expert insights from the workshops held as part of the OECD NanoAOP project. Limitations identified through these efforts include challenges specific to MNs, as well as outstanding research and technical needs hindering AOP development more generally.

### 6.1. Nanomaterial-Specific Challenges

The development and application of AOPs for MN decision making pose a specific set of challenges, summarized as follows:

#### 6.1.1. Limitations of Current Literature 

Although there have been significant advancements in nanosafety and nanotoxicology research over the last two decades [30], there are several limitations of the literature for AOP development and use. Limitations include (i) a lack of complete understanding of the biological mechanisms of action underlying MN-induced adverse health effects; (ii) uncertainty from the different exposure conditions and models (e.g., assays, cell lines, etc.) used in each study; (iii) limited consideration of MN dispersion and dosimetry; (iv) a general lack of physical and chemical characterizations of MNs (see *Influence of Physical and Chemical Properties,* below); and (v) fragmentation of the data [31,32,33]. Better data creation and management processes for MNs are required to advance the development and use of the AOP framework, and future data reporting needs to include a set of minimum information requirements [31], although, as noted in Box 2, there are efforts underway to address this challenge.

Box 2 **Projects contributing to the development, use and adoption of AOPs for MNs.**

**SmartNanoTox** Conducting in vivo, in vitro and in silico research to develop AOPs for adverse pulmonary effects following MN exposure. Research is being used to develop simplified in vitro or in silico tests for the AOPs developed in the project, targeting identified MIEs and KEs for adverse respiratory outcomes from MN inhalation.**PATROLS** Developing mechanism-based, nonanimal methods, models and computational tools for MN hazard characterization, targeting the KEs in established AOPs. This includes in silico hazard testing systems, in vitro human tissue models, ecotoxicology models and methods for MN characterization in biological systems.**OECD WPMN NanoAOP** Developed a methodology and approach to use existing nanotoxicology literature to support MN-relevant AOP development and its use in decision making.**NanoSolveIT** Developing: (i) innovative modeling techniques and tools for nanoinformatics; (ii) an IATA to identify the specific characteristics of MNs that are responsible for adverse effects on human health or the environment; and (iii) in silico methods, models and tools which are useful for AOP development using toxicogenomics data and linked to nanomaterial “fingerprints”. See also [19].**NanoCommons** Developing a nanoinformatics research infrastructure including a knowledge base to facilitate the reuse of existing nanosafety data, tools to support Open and FAIR data curation and annotation, and in silico tools for analysis and prediction of MN environmental and human health impacts.**GRACIOUS** Developing a grouping strategy for MNs that can be incorporated into an IATA.**COSMOS** Identifying common sets of metadata objects with standard definitions and methods to build better metadata repositories.**DaNa** A database with important and generally understandable information on health and the environment as they relate to the application of nanomaterials, as well as data on the safety of manufactured nanomaterials.**RiskGONE** Developing science-based risk governance of MNs based on an understanding of risks and risk management practices. The project is developing new tools and/or modifying existing ones to identify the environmental and human health impacts of MNs. AOPs for human and environmental end-points are being developed. These tools will be integrated into the work of a European Risk Governance Council to provide governance decisions on the safety of the specific materials.**NanoReg2** Aimed to couple ‘safe-by-design’ (SbD) to the regulatory process, using value chain implementation studies to establish SbD as a fundamental pillar in the validation of a novel NMs. Grouping concepts developed by NanoReg2 were prepared as guidance documents to support industries or regulatory agencies.

#### 6.1.2. Assays and Methods to Assess MIEs and KEs 

To use AOPs for decision making about MNs, there is a need to identify which KEs are critical for testing as part of an IATA. To apply AOPs to chemical risk assessments, strategies to evaluate specific MIEs and KEs must be developed and verified [7]. Many of these assays and testing strategies exist for conventional chemicals, but toxicity testing of MNs often requires modification [34,35,36,37]. Method development, including verification of MN-appropriate methods, is needed, and remains an on-going challenge, although significant progress in being made. Highlighted needs specific to MNs arising from expert discussions include the incorporation of accurate dosimetry into toxicity testing, which includes consideration of the most relevant dose metrics (e.g., mass, surface area, or particle number) and exposure conditions (e.g., stability of suspensions and characterization of agglomerates) [38]. In developing assays and methods to assess MIEs and KEs, reproducibility and accessibility to testing methods need to be considered. Further, assays and methods will have to be evaluated for their relevance to a given AOP; some assays may be relevant to more than one AO. Multiple assays may be required to evaluate KEs and ensure specificity to a given AOP (e.g., assays looking at cytotoxicity via different mechanisms, such as apoptosis versus necrosis). A key gap for both chemicals and MNs is the development of AOPs for ecotoxicity, a topic that is being addressed in RiskGONE and NanoSolveIT.

#### 6.1.3. Influence of MN Physical and Chemical Properties

Traditional chemical properties such as solubility, hydrophobicity and chemical composition are known to influence the toxicity of MNs. However, MN toxicity can also be influenced by distinct physical–chemical properties, such as dissolution rate (ion release), electronic band gap, aspect ratio, dispersibility in solution, large surface area to volume ratios or increased surface reactivity [39]. AOPs are generally developed with data from conventional, bulk chemicals; their application to MN risk assessment requires evaluation to ensure that MN-relevant mechanisms, MIEs and KEs are captured [9,11]. In particular, workshop experts identified that one issue facing MN-relevant AOP development is the lack of identified MIEs. For MNs, MIEs may be physical rather than molecular in nature (e.g., frustrated phagocytosis); research and AOP development will need to account for these particle-specific mechanisms. Currently, there are nine OECD-endorsed AOPs, seven that have received approved status, fourteen under review, and twenty-four proposals under active development, which could be assessed for their relevance to MNs.

Despite these challenges, workshop experts concluded that the AOP framework would improve risk assessment strategies for MNs, and further the community’s understanding of toxicity mechanisms and potency. Realizing this potential requires addressing outstanding technical challenges and barriers to adoption, i.e., both those specific to MNs (described above) and those that apply to AOPs more generally.

### 6.2. Technical Challenges

#### 6.2.1. AOP Networks 

Individual AOPs are constructed as linear sequences of biological events connecting a MIE to an AO. However, exposure to chemicals, including MNs and other emerging substances, may affect more than one MIE or KE, and result in one or many AOs. Individual AOP units are intended to form networks of interconnected KEs to reflect this complexity, but little guidance is currently available to develop, analyze and evaluate these networks [9].

#### 6.2.2. Exposure and Dose 

Risk assessment requires information on the exposure conditions (e.g., route, dose, duration and frequency) needed to cause an AO. Quantitative AOPs (qAOPs), which use quantitative data to predict risk of an AO under specific exposure conditions, are proposed as a solution to address these needs, but few examples currently exist.

#### 6.2.3. Individual and Interspecies Differences 

Additional challenges include how to develop AOPs that can account for individual variation, such as life stage, immune status or sex, and how to reduce the uncertainty that arises from interspecies differences, including sensitivity, potency and metabolic diversity.

#### 6.2.4. Repair Mechanisms

Many key biological events, although triggered with exposure, can resolve over time and do not result in an AO. Such repair mechanisms may not be accounted for in the AOP framework, and identifying ‘points of no return’ toward an AO is important for using the AOP framework for decision making. Consideration of dose-response (i.e., exposure) within the framework would help account for repair mechanisms.

### 6.3. Barriers to Adoption

#### 6.3.1. Lack of Guidance for Risk Assessors 

Guidance is needed for risk assessors outlining the use and application of AOPs for decision-making. Such guidance should help risk assessors determine whether the level of development for an AOP and weight of evidence provided when using it as part of an IATA are adequate for decision-making. The emerging consensus is that the level of understanding and degree of confidence needed will depend on the intended application and severity of the AO, i.e., whether an AOP is ‘fit-for-purpose’ must be evaluated on a case-by-case basis. For example, for AOPs with more severe outcomes (e.g., fibrosis or cancer), an IATA would likely require a battery of validated tests spanning several KEs in the pathway to establish the weight-of-evidence needed for decision-making. Further, negative results would require a higher burden of proof. Establishing guidance on this topic would help accelerate AOP adoption.

#### 6.3.2. Engagement of Multiple Stakeholders

The development of AOPs requires a significant investment of resources, time and expertise. Engagement of multiple stakeholders with a broad range of expertise is essential; coordination and cooperation are needed for efficient, high-quality AOP development. Multistakeholder participation is critical to increase confidence in the framework and to support its transition toward use in policy, decision making and regulatory applications, but challenges exist in engaging participants. These include, among others, a lack of adequate incentives, problems with information control and ownership, reputational and liability risks associated with developing AOPs for decision making purposes, resource demands and limitations, cross-discipline communication challenges and the need for oversight [2].

#### 6.3.3. Communication

Even among experts, misconceptions about the AOP framework exist. In the horizon scanning effort, community collaboration and communication were identified as critical components of AOP development and acceptance.

## 7. Summary

The adoption of AOPs for advanced materials and MNs is hindered by the lack of accepted methods for toxicity evaluation and over-reliance on resource-intensive animal testing. There is a need and an opportunity to develop smarter approaches for screening and prioritizing novel nanoscale materials for decision-making. The AOP framework is a promising and toxicologically realistic approach to help address several current challenges of chemical and nano-specific safety assessments. Significant progress has been made in AOP development, application and use over the last decade including methods, tools and assessment approaches. However, we are still a long way from reliance on—and the replacement of animal testing with—nonanimal approaches. Several challenges remain and must be addressed to realize the full potential of AOPs. Projects worldwide are focusing on meeting these challenges and advancing the use of AOPs for decision-making. International cross-disciplinary analyses and deliberation will improve the adoption and acceptance of the AOP framework, including for MNs.

## 8. The Way Forward

To advance the development, use and acceptance of the AOP framework for risk assessment and decision making, and to help overcome the challenges identified above, several actions are suggested which aim at promoting nine central recommendations (Table 3). The ‘central recommendations’ (Table 3) are meant to be high-level goals, with the ‘actions’ the means of achieving them, broken out by timing. Although these recommendations and corresponding actions are tailored to promoting the development, use and acceptance of AOPs, they share overlapping goals with the nanosafety research community at large. Actions addressing these central recommendations are organized by timelines expected for completion: short-, medium- and long-term. 

## Figures and Tables

**Table 1:** Discussion themes posed to OECD workshop participants.

**1. How can the use of the AOP framework be advanced for decision making about the safety of MNs?**
**2. What is needed for the future development of MN-relevant AOPs and supporting data?**
**3. How can the development and adoption of in vitro assays be targeted to KEs to enable the use of AOPs as part of an IATA for MN decision making?**

**Table 2:** Potential applications and benefits of the AOP framework in chemical risk assessment.

1. Evaluation of existing information
improve chemical grouping and categorization to fill data gaps by read-across
utilize data developed from advances in alternative testing strategies, such as in chemico, in vitro, ex vivo and ‘omic’ data, for decision making
improve predictivity in safety assessment (e.g., quantitative structure–activity relationships)
2. Identification of data gaps and generation of new data
improve high-throughput screening for chemical prioritization
identify data gaps to inform relevant research
identify novel biomarkers for hazard evaluation
develop novel, nonanimal approaches for hazard characterization
reduce reliance on animal testing
3. Iterative decision making
increase confidence in nontraditional test methods
use nontraditional data and methods to improve weight-of-evidence in decision making

**Table 3:** Central Recommendations for Promoting the Development, Use and Acceptance of the AOP Framework for MN Decision Making.

**1. Advance MN-relevant and Advanced Material Considerations in AOP Development** The AOP framework requires the continued development of predictive pathways, building on the efforts of the toxicology community via the AOP Wiki. Needs include updates to toxicological mechanisms within AOPs that identify processes and considerations relevant to MN toxicity. Further, the path for emerging advanced and hybrid materials to use the AOP Framework for early-stage safety decisions can be outlined from these efforts. Recommendations include:		
*Short-term Actions*	*Medium-term Actions*	*Long-term Actions*
1. Establish the types of data required to develop AOPs for MNs and identify existing NM-relevant AOPs; 2. Compare molecular initiating events (MIEs), key events (KEs), key event relationships (KERs) and adverse outcomes (AOs) identified for MNs to AOPs in the AOP Wiki; 3. Identify MN-relevant MIEs, KEs and KERs. Research should include identifying MN-relevant mechanisms and MIEs, which are often based upon physical interactions with MNs instead of molecular ones (e.g., frustrated phagocytosis and particle-surface-induced reactive oxygen species [9]).	1. Identify similarities and differences in MIEs, KEs and KERs between other emerging advanced materials and MNs [9]; 2. Conduct targeted research on MNs to elucidate the effect of interspecies variability on AOPs and the development of related testing strategies [40,41]. This can include side-by-side testing of in vitro cell lines and 3D models from a number of species exposed to a suite of MNs to examine conserved mechanisms and potencies; 3. Use ‘omics’ approaches to identify gene, protein and metabolite markers of MN exposure (e.g., using heatmaps) and their implications for AOP development [24,39,42].	1. Develop data sets for quantitative AOPs (qAOPs) that include consideration of the exposure conditions necessary for MN risk assessment. This includes adopting formal definitions and structures for qAOPs and developing case studies outlining the development and use of qAOPs for MNs; 2. Develop a testing strategy for advanced and hybrid materials (smart and responsive materials) to identify and quantify MIEs, KEs, KERs and AOs [9]; 3. Convene experts to discuss how AOPs can account for individual (e.g., sex or life-stage) and interspecies variations which can be then used to reduce the associated uncertainty for decision making.
**2. Utilize Existing Data from the Literature and Previous Projects** A diverse set of data has been developed that may be useful for furthering the development, application and use of AOPs for MN risk assessment. So far as possible, these data should be taken advantage of to advance knowledge and identify opportunities for additional AOP development. This requires extensive expert-driven curation efforts. Recommendations include:		
*Short-term Actions*	*Medium-term Actions*	*Long-term Actions*
1. Evaluate data quality from the peer review literature and current suite of in vitro assays based on identified KEs; 2. Encourage researchers (and publishers) to make their raw toxicology data from peer-reviewed literature available publicly; 3. Harmonize formats for reporting toxicology data (including negative results) to facilitate the development of databases; 4. Build searchable databases for priority MNs that includes funded research and literature (e.g., available data developed under NanoCommons, eNanoMapper, NanoReg2, GRACIOUS and DaNa projects), as well as traditional chemical databases (e.g., TOXCAST). Efforts should include collecting negative data.	1. Develop guidance on how the existing nanotoxicity literature, despite its documented limitations (e.g., minimal reporting of physical and chemical characteristics), can be used for AOP development and decision making; 2. Broaden access and use of existing data sources (e.g., Nanomaterial-Biological Interactions Knowledgebase, Nanomaterials Knowledge Informatics Commons (NIKC); eNanoMapper, NanoCommons KnowledgeBase) and other resources; 3. Evaluate publicly available REACH data for MNs in terms of use in AO and predictive modeling; 4. Identify novel biomarkers for hazard evaluation; 5. Develop research projects to fill data gaps for identified endpoints.	Create processes to continually update publicly available databases as new data is developed.
**3. Promote Reliable and Quantitative MN Data Generation and Management** High quality data are essential to ensure that MN-relevant AOPs can be developed and used in decision making. Guidance is needed on the types of data and reporting standards to enable the use of AOP in regulatory decision making. Coordinated efforts among stakeholders will improve efficiency and limit additional testing. Recommendations include:		
*Short-term Actions*	*Medium-term Actions*	*Long-term Actions*
1. Identify priority AOs observed with MNs and initiate research into AOP development for these AOs; 2. Standardize the endpoints and reporting elements of assays evaluating MIEs, KEs and KERs so that high quality, comparable data is generated, published and added to databases, including negative data; 3. Develop guidance on the types of data that need to be generated and reported by the research community for their work to be useful in regulatory decision making.	1. Generate data to allow for grouping – data collection/mining to determine the mode of action using MNs that can represent groups of MNs/functionalizations; 2. Develop MN-specific resources (for the AOP Wiki) to encourage the coordination and cooperation among stakeholders which is needed for efficient, high-quality AOP development.	1. Advance modeling and QSAR databases and link to the physical and chemical attributes of MNs; 2. Adopt iterative decision making, including increased confidence in nontraditional methods and use of nontraditional data and methods to improve weight-of-evidence in decision making.
**4. Advance Knowledge of the Quantitative Relationships Between MN Physical and Chemical Characteristics and AOP Elements** A better understanding of the quantitative relationships between MN physical and chemical characteristics and toxicological outcomes is required. It is recommended to:		
*Short-term Actions*	*Medium-term Actions*	*Long-term Actions*
1. Review findings of existing data and research on the relationships between physical and chemical properties and MN KEs, including MIEs, AOs and KERs; 2. Develop hypotheses of predictive relationships between MN physical and chemical properties and biological outcomes.	1. Test predictive physical and chemical relationships of MNs to biological outcomes, using carefully controlled changes within and across materials (furthering the work of the projects which inaugurated this effort, such as SmartNanoTox and NanoMILE); 2. Assess the importance of using alternative dose metrics to mass (e.g., surface area, particle number) in predicting toxicological outcomes for MNs; 3. Where appropriate, incorporate alternative dose metrics into developed benchmark levels for MNs for screening and risk assessment.	1. Develop quantitative structure–activity relationships (QSAR) as predictive tools for KEs.
**5. Identify Current Applications of the AOP Framework for MN Decision Making** Current applications of the AOP framework (e.g., prioritization, grouping and read-across) can be adopted into decision making. It is recommended to:		
*Short-term Actions*	*Medium-term Actions*	*Long-term Actions*
1. Identify screening-level MN safety decisions that are fit-for-purpose/can rely on AOPs; 2. Incorporate AOP elements into grouping and read-across decision trees for MNs.	1. Adopt a testing scheme/decision tree for MN grouping and read-across; 2. Develop guidance and case studies for use of AOPs in regulatory decision making (e.g., MN prioritization; grouping, categorization and read-across; and hazard identification and ranking).	1. Develop guidance and case studies for the use of AOPs in product development decision making, and the implementation of a safe-by-design approach.
**6. Establish Test Methods and Protocols which are Useful for MN Decision Making** Test methods to accurately measure MN-relevant MIEs and KEs are required to advance the use of AOPs as part of an IATA for MN decision making. The development (and verification) of harmonized and standardized MN-relevant test methods is needed:		
*Short-term Actions*	*Medium-term Actions*	*Long-term Actions*
1. Evaluate, advance or develop physical and chemical characterization protocols for MNs and determine how they can be used to identify MIE, KE and AO portions of the AOP framework; 2. Prioritize KEs and the assays/methods to characterize them for development, with KEs closer to an AO being prioritized to ensure relevance for regulatory decision making as part of an IATA; 3. Evaluate, advance or develop in silico, in chemico, in vitro and ex vivo assays for MNs and determine how they can be used to characterize MIE, KE and AO portions of the AOP framework [24]; 4. Initiate Test Guideline development for assays tailored to MNs that address considerations such as physical and chemical characterization, dispersion and dosing relevant to AOPs; 5. Develop guidance on the minimum level of validation required for a given in vitro assay or method for regulatory decision making.	1. Create voluntary standard methods for IATA; 2. Consider the formal adoption of IATA for certain MN hazard or risk decisions; 3. Advance new in vitro test development to screen for MIEs and KEs; 4. Identify test methods (including in silico) for high throughput screening.	1. Develop OECD test guidelines for MNs that relate to MIEs, KEs and AOs; 2. Where appropriate, formally adopt IATA for MNs; 3. Adopt harmonized, standardized tests for high throughput screening.
**7. Demonstrate Predictive Capability of AOPs and In Vitro Test Methods** A coordinated effort is needed to ensure alternative testing strategies are predictive of adverse outcomes of regulatory relevance. Recommendations include:		
*Short-term Actions*	*Medium-term Actions*	*Long-term Actions*
1. Assess the strength of evidence for considering dose-response relationships in AOPs as predictive tools for MN risk assessment.	1. Compare the predictive capability of in vitro assays for MIEs and KEs with in vivo observations or epidemiological data; 2. Design and conduct side-by-side in vitro and in vivo testing for representative MNs to compare toxicity mechanisms and potency across MNs and assays (furthering the work of the projects that inaugurated this effort, such as PATROLS).	1. Develop and test predictive alternative testing models; 2. Validate predictive alternative testing models.
**8. Guidance to Facilitate Adoption of MN-relevant AOPs for MN Decision Making** The science required to address the technical challenges of transitioning to alternative (i.e., nonanimal) toxicity testing is progressing, but efforts are needed to translate and incorporate these developments into decision making about the safety of MNs. Recommendations include:		
*Short-term Actions*	*Medium-term Actions*	*Long-term Actions*
1. Identify MN-relevant and MN-specific AOPs, KEs and KERs, including assessments of the AOPs which have been officially OECD-endorsed, approved or are under review and under active development for MN-relevance (e.g., https://aopwiki.org/aops/173)	1. Develop and validate an IATA based on KEs, KERs and AOPs that can be used in risk assessments of new nanoscale materials. This includes identifying and prioritizing which KEs are critical for testing as part of an IATA, building on the work currently ongoing in NanoSolveIT; 2. Develop guidance for risk assessors on developing an IATA based on AOP frameworks for MN safety assessments. This should include how to pick critical KEs, or a suite of KEs, for testing.	1. Incorporate technical developments into specific regulatory guidance/policy documents.
**9. Stakeholder Communication and Engagement on the Use of AOPs for MN Decision Making** To facilitate the development, adoption and use of the AOP framework for MN decision making, the engagement of multiple stakeholders with a broad range of expertise is essential, and coordination and cooperation are needed. Recommendations include:		
*Short-term Actions*	*Medium-term Actions*	*Long-term Actions*
1. Develop communication and educational materials on the use of the AOP framework for MN decision making for nontechnical stakeholders; 2. Organize additional workshops which seek to encourage participation from various stakeholders with a vested interest in AOP development and application for MN decision making, including academics, policy-makers, regulators and industry.		
